# *Sarcocystis neurona* Transmission from Opossums to Marine Mammals in the Pacific Northwest

**DOI:** 10.1007/s10393-021-01536-w

**Published:** 2021-07-02

**Authors:** Alice M. O’Byrne, Dyanna M. Lambourn, Daniel Rejmanek, Katherine Haman, Michael O’Byrne, Elizabeth VanWormer, Karen Shapiro

**Affiliations:** 1 School of Veterinary Medicine, University College Dublin, Belfield, Dublin, D04 W6F6 Ireland; 2 Wildlife Program, WA Department of Fish and Wildlife, 1111 Washington Street SE, Olympia, WA 98501 USA; 3 California Animal Health and Food Safety Laboratory, Davis, CA USA; 4 University of Göttingen, Wilhelmsplatz 1, 37073 Göttingen, Germany; 5 School of Veterinary Medicine and Biomedical Sciences, School of Natural Resources, University of Nebraska-Lincoln, Lincoln, NE USA; 6 Pathology, Microbiology and Immunology, University of California Davis, One Shields Avenue, 4206 VM3A, Davis, CA 95616-5270 USA

**Keywords:** Sarcocystis neurona, Marine mammals, Transmission, Watershed, Groundwater, Opossums

## Abstract

Increasing reports of marine mammal deaths have been attributed to the parasite *Sarcocystis neurona*. Infected opossums, the only known definitive hosts, shed *S. neurona* sporocysts in their feces. Sporocysts can contaminate the marine environment via overland runoff, and subsequent ingestion by marine mammals can lead to fatal encephalitis. Our aim was to determine the prevalence of *S. neurona* in opossums from coastal areas of Washington State (USA) and to compare genetic markers between *S. neurona* in opossums and marine mammals. Thirty-two road-kill opossums and tissue samples from 30 stranded marine mammals meeting inclusion criteria were included in analyses. Three opossums (9.4%) and twelve marine mammals (40%) were confirmed positive for *S. neurona* via DNA amplification at the ITS1 locus. Genetic identity at microsatellites (sn3, sn7, sn9) and the snSAG3 gene of *S. neurona* was demonstrated among one harbor porpoise and two opossums. Watershed mapping further demonstrated plausible sporocyst transport pathways from one of these opossums to the location where an infected harbor porpoise carcass was recovered. Our results provide the first reported link between *S. neurona* genotypes on land and sea in the Pacific Northwest, and further demonstrate how terrestrial pathogen pollution can impact the health of marine wildlife.

**Supplementary Information:**

The online version contains supplementary material available at 10.1007/s10393-021-01536-w.

## Introduction

*Sarcocystis neurona* is a protozoan parasite with a life cycle that includes both definitive and intermediate hosts. Intermediate hosts include equines, felines and marine mammals such as sea otters (*Enhydra lutris)*, harbor seals (*Phoca vitulina)*, harbor porpoise (*Phocoena phocoena*), sea lions (*Eumetopias jubatus)*, pygmy sperm whales *(Kogia breviceps)* and Pacific white-sided dolphins (*Lagenorhynchus obliquidens)* (Mullaney et al. 2005; Dubey et al. 2000; Barbosa et al. 2015). The opossum (*Didelphis virginiana*), the only known definitive host for *S. neurona*, consumes sarcocysts via ingestion of infected intermediate host tissue. Sexual replication of the parasite in the intestinal tract of opossums leads to formation of oocysts that contain 2 sporocysts. Oocysts and sporocysts are then passed into the environment in feces (Dubey et al. 2001; Fenger et al. 1995).

In intermediate hosts, *S. neurona* can encyst in muscle or central nervous system (CNS) tissue without causing clinical signs (Dubey et al. 2001). However, encephalitis can result in some animals infected with *S. neurona*, with strain type likely mediating the virulence of this pathogen (Barbosa et al. 2015; Wendte et al. 2010b). *Sarcocystis neurona* is a causative agent of equine protozoal myeloencephalopathy (EPM) (Dubey et al. 1991) and has been increasingly implicated in marine mammal mortality (Thomas et al. 2007; Miller et al. 2010; Barbosa et al. 2015). For the related protozoan parasite, *Toxoplasma gondii,* several studies have suggested that oocysts can become mobilized via overland runoff into coastal waters, with subsequent concentration in marine invertebrates that can serve as a source of infection to marine mammals (Arkush et al. 2003; Miller et al. 2004; Conrad et al. 2005; Shapiro et al. 2012, 2014; Schott et al. 2016). Studies of ground and surface water has confirmed the presence of *T. gondii* DNA (Bahia-Oliveira et al. 2017).

Of particular importance is the fatality associated with *S. neurona* infection in federally listed species such as the threatened Southern sea otter (*Enhydra lutris nereis*) (Kreuder et al. 2003; Miller et al. 2010, 2018). In 9.8% of sea otters *S. neurona* infection was attributed as the primary cause of death (Thomas et al. 2007). In Washington State (USA), *S. neurona* was recently identified as the leading cause of death in Northern sea otters (*Enhydra lutris kenyoni*), with 30% of animals (*N* = 93) examined between 2002 and 2015 dying due to *S. neurona* encephalitis (White et al. 2018). An earlier investigation demonstrated that 61.5% (*n* = 161) of marine mammal carcasses in the Pacific Northwest were infected with *S. neurona*, and the relative frequency of these infections had increased from 2004 to 2009 (Gibson et al. 2011).

The overarching goal of this study was to investigate the molecular epidemiology of *S. neurona* from terrestrial to marine mammals in the Pacific Northwest. Specifically, our aims were to (1) analyze gastrointestinal (GI) scrapings from opossums from western Washington State to evaluate the prevalence of animals shedding *S. neurona*; (2) test marine mammal tissues for the presence of *S. neurona* infection; and (3) use molecular signatures to compare *S. neurona* strains present in opossums and marine mammals in this region.

## Materials and Methods

### Marine Mammal and Opossum Sample Collection

**Figure 1:**
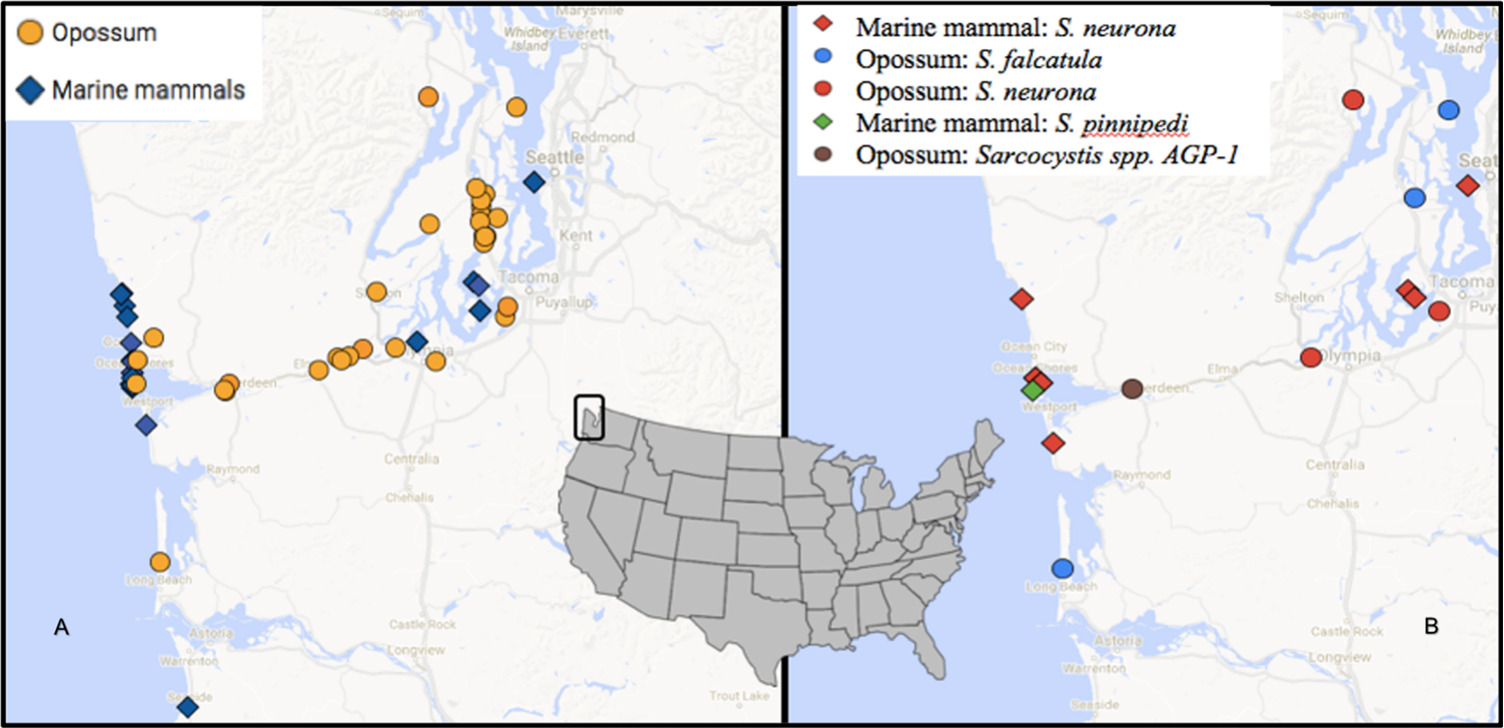
Map detailing the sampling sites of animals collected from western Washington State, USA between 2015 and 2017. **a** Location of all marine mammals (blue diamonds) and opossums (orange circles) analyzed for the presence of *Sarcocystis* spp. DNA. **b** Confirmed *Sarcocystis* spp. infections in marine mammals (diamonds) and opossums (circles). Inset map indicates the sampling area within the USA.

Tissues from 30 marine mammals were collected from animals that stranded along coastal regions of Washington State, USA, between 2015 and 2017 (Fig. 1). Carcasses were opportunistically collected by the Washington Department of Fish and Wildlife (WDFW) and the Cascadia Research Collective as part of a coastal health monitoring program by the West Coast Marine Mammal Stranding Network [Marine Mammal Protection Act Stranding Agreements and Section 109(h), 16 U.S.C 1379(h)]. Tissue samples that were collected included brain, skeletal and cardiac muscle, liver and lymph node. Marine mammal species, collection dates, location, tissue type analyzed, live stranding and histopathological categorization are listed in Table S1. Pregnant marine mammals that had unborn fetuses are indicated as pairs with the suffix “F” denoting the fetus. Marine mammal inclusion criteria for this study were (1) confirmed or suspected protozoal encephalitis based on previous histopathological analysis; (2) evidence of protozoal infection outside of the central nervous system; (3) live stranding events with evidence of neurological abnormalities; and (4) carcasses for which histopathology was not available if animals were recovered from areas where recent confirmed encephalitis cases were encountered or during months when increased cases of protozoal encephalitis have been previously described (April–June; White et al. 2018).

Opossum carcasses were opportunistically collected following identification of individual animals killed along roadways in coastal Washington by the WDFW and Cascadia Research Collective. Carcasses were frozen at − 20°C and thawed at 4°C for 48 h prior to processing. Carcasses were dissected and the gastrointestinal tract was exposed. The small intestine was removed distal to the pylorus and proximal to the caecum. Working in 3-inch segments, the intestine was flushed of ingesta using phosphate-buffered saline (PBS), cut lengthwise and the inner mucosal layer was scraped with the edge of a glass slide. GI scrapings were collected in a sterile 15-ml container and shipped on dry ice from the WDFW to the University of California, Davis for molecular analysis.

### Opossum GI Scrapings: Sporocyst Visualization and Molecular Identification

GI scrapings were examined via direct brightfield microscopy at 400× magnification for the presence of sporocysts. Scrapings containing sporocysts were washed in 10 ml PBS three times (1000×g 10 min), agitated in 2.6% sodium hypochlorite for five minutes, followed by three additional PBS washes (Rejmanek et al. 2009). The resulting pellet was frozen at − 20°C until further processing.

For DNA extraction, sporocyst pellets were initially subjected to one freeze–thaw cycle performed via 5 min immersion in liquid nitrogen followed by 5 min in boiling water. A Qiagen™ tissue extraction kit (Qiagen, Valencia, CA) was used to extract nucleic acids, following manufacturer recommendations, with the exception of adding 40 µl proteinase K in the initial incubation. For DNA amplification, a nested primer set (ITS1-500) targeting a 500 bp fragment of the Internal Transcribed Spacer 1 (ITS1) gene, which is specific for *Sarcocystis falcatula* and *S. neurona* but does not amplify other *Sarcocystis* spp. or *T. gondii,* was used (Miller et al. 2009). Primer sequences are listed in Table S2, and PCR conditions were followed according to the references listed for each primer set. PCR products were separated on a 1.5% agarose gel stained with ethidium bromide and visualized using ultraviolet light. Amplification products consistent in size with a *S. neurona*-positive control sample were excised and DNA was purified with Qiagen™ QIAquick Gel extraction kit (Qiagen, Valencia, CA) following manufacturer instructions. Purified DNA samples were submitted to the University California Davis Sequencing Facility. For sequence analysis, the forward and reverse sequences were aligned (Geneious software, Biomatters, Auckland, New Zealand), ends trimmed and the consensus sequence compared with GenBank reference sequences using BLAST (http://blast.ncbi.nlm.nih.gov/Blast.cgi).

### Marine Mammal Tissues: Nucleic Acid Extraction and Amplification

Marine mammal tissues were dissected and placed into a 1.5-ml Eppendorf tube. DNA extraction was performed using the Qiagen™ tissue extraction kit (Qiagen, Valencia, CA) following manufacturer instructions. The extracted DNA was amplified using the IST1-500 assay as described above, and/or a less specific nested primer set targeting the ITS1 region that can co-amplify other protozoan parasites, including other *Sarcocystis* species and *T. gondii* (Miller et al. 2009). Due to fiscal constraints, 10 samples were tested using the ITS1-500 assay, and 20 samples were tested using the ITS1 assay—providing data on both *Sarcocystis* and *T. gondii* prevalence in these 20 samples. PCR amplification products consistent with positive controls [*S. neurona* (~ 1100 bp) or *T. gondii* (~ 500 bp)] were excised, purified and submitted for sequence analysis as described above.

### Molecular Characterization of S. neurona

Samples that were confirmed as *S. neurona* via sequence analysis were further characterized using four genetic markers (Rejmanek et al. 2009, 2010). The selected markers included one surface antigen gene (snSAG3) and three microsatellite markers (sn3, sn7 and sn9) that have shown moderate-high polymorphism in previous investigations (Rejmanek et al. 2010). Primer sequences and thermocycler conditions for each assay are listed in Table S2. The number of dinucleotide repeats was recorded for each of the microsatellites, while for snSAG3 analysis, the nucleotide positions which differed from a *S. neurona* consensus sequence (GenBank sequence GQ386977) were identified as single nucleotide polymorphisms (SNPs).

### Watershed Delineation and Groundwater Contours

Basic hydrogeological mapping was performed to establish a conservative waterborne transport pathway from land to sea. For this modeling scenario, we assumed that *S. neurona* sporocysts have similar environmental transport properties as *Toxoplasma gondii* oocysts, a related apicomplexan parasite known to travel in both groundwater and surface water (Bahia-Oliveira et al. 2017). Both groundwater and surface water mapping were performed as *T. gondii* has been shown to be present in both runoff and groundwater sources (Bahia-Oliveira et al. 2017). Digital elevation maps (DEM) were obtained for the study area using earth explorer (https://earthexplorer.usgs.gov/). Multiple DEM images were required to sufficiently cover the study area, and separate DEMs were combined using Qgis 3.2.3-Bonn (QGIS 2019). The combined DEM was projected into NAD83 Universal transverse Mercator (UTM) zone 10 N. The Projected DEM was manipulated using the GRASS GIS (GRASS 2017) toolset by removal of sinks, generation of a flow accumulation raster and water outlet to delineate watersheds. Sinks in the DEM were filled using GRASS r.fill flow accumulation and direction raster was generated using GRASS r.watershed resulting in river course map and watershed. Lastly, using the flow direction raster, the GRASS r.water.outlet tool was used to determine the water outlet point of the generated watershed.

Considering the proximity of opossum OP1 to an area of elevated land which separates two watersheds and that the flow of surface water is not necessarily tied to groundwater gradients, it was prudent to generate a groundwater contour map. The groundwater map was generated using data from the USGS groundwater monitoring program (U.S. Geological Survey 2018). A USGS dataset from summer 2016 was selected due to temporal proximity to the study period and good spatial coverage. Using the bore locations in UTM (X, Y) and elevation (feet, Z), an X,Y,Z grid was generated representing the groundwater surface. The surface grid was generated using a kriging method in surfer 15 (Golden Software, Golden, CA).

## Results

### Marine Mammals

The species of stranded marine mammals included in the study are summarized in Table 1. Figure 1 depicts the stranding location of marine mammals that tested positive for *Sarcocystis* spp. Sequence analysis confirmed *S. neurona* infections in 12 marine mammals, yielding an overall prevalence of 40% (Table 1 and Table S1). In two northern sea otter samples, SO4 and SO3, DNA amplification yielded products consistent in size with *Sarcocystis*, however, sequencing failed, and thus no molecular confirmation was possible. Therefore, SO4 and SO3 were not reported as *S. neurona-*infected marine mammals in this study. In one harbor seal (HS7), DNA amplified via the ITS1 assay was determined to have 100% identity with *Sarcocystis pinnipedi* (GenBank No. MT460246) (Haman et al. 2013; Miller et al. 2018). Simultaneous protozoal infections in dam and neonate were observed in a sea lion 1 (SL1) and her in-utero fetus (SL1F) with *S. neurona*, and in HP4 (dam) and HP4F (fetus) with *T. gondii*. Of the 20 animals tested with primers that could identify *Sarcocystis* and *T. gondii*, one harbor porpoise (HP4) was co-infected with *S. neurona* and *T. gondii*, yielding a 5% prevalence of dual protozoan infection. Overall, 4 marine mammals (HP4, HP4F, SO3, DP1) were infected with *T.* gondii (20%% prevalence) (Table S1).

**Table 1:** Prevalence of *Sarcocystis neurona* in Opossums and Marine Mammals Sampled in Western Washington State, USA between 2015 and 2017

Animal type	Scientific Name	Common name	No. of Animals	No positive for *S. neurona* (prevalence)	Other *Sarcocystis spp* found	Tissue Tested^a^
Opossum	*Didelphis virginiana*	Opossum	32	3 (9.4%)	*S. falcatula* (*n* = 4) *S.* AGP-1 (*n* = 1)	GIS
Marine Mammal	*Eumetopias jubatus*	Steller sea lion	3	2 (66.6%)		B
	*Enhydra lutris*	Northern sea otter	5	1 (20%)		B
	*Pusa hispida*	Ringed seal	1	0 (0%)		B
	*Phoca vitulina*	Harbor seal	11	3 (27%)	*S. pinnipedi*^b^ (*n* = 1)	B, M (*n* = 2)
	*Phocoena phocoena*	Harbor Porpoise	6	4 (66.6%)		B
	*Arctocephalus townsendi*	Guadalupe fur seal	1	0 (0%)		B
	*Phocoenoids dalli*	Dall porpoise	1	0 (0%)		B
	*Delphinus capensis*	Long-beaked common dolphin	1	1 (100%)		B
	*Lagenorhynchus obliquidens*	Pacific white-sided dolphin	1	1 (100%)		B

^a^GIS = gastrointestinal scraping, B = brain tissue, M = skeletal muscle

^b^GenBank accession no. MT460246

### Opossums

Figure 1 depicts the location of opossums that tested positive for *Sarcocystis* spp. Of the 32 opossums, 9 had sporocysts visualized in GI scrapings using microscopy. Nucleic acid amplification products from three (9.4%) of these samples were confirmed via sequence analysis as *S. neurona*, four (12.5%) were confirmed as *Sarcocystis falcatula* and OP21 contained sporocysts that yielded a DNA sequence consistent with *Sarcocystis sp.* AGP-1 (GenBank accession no. DQ768306.1; 100% identity). In one sample containing sporocysts (OP5), sequencing attempts failed, and thus this sample was not included in further genetic characterization.

### Molecular Characterization of S. neurona

Further molecular characterization of *S. neurona* was performed in the 12 marine mammals and 3 opossums that were confirmed to be infected. Single nucleotide polymorphisms (SNPs) in one polymorphic gene (SnSAG3) and variations in dinucleotide repeats in three microsatellite markers are reported in Table 2. Sequence analysis of the snSAG3 gene demonstrated seven SNPs at nucleotide position 239, 506, 507, 508, 509, 735 and 1057. Dinucleotide repeats at the sn9 microsatellite showed little polymorphism, with only two variants, while sn7 had the highest variability with four variants. Genetic identity was confirmed across these four genetic markers among two opossums (OP1 and OP3) and one harbor porpoise (HP2).

**Table 2:** Molecular Characterization of *Sarcocystis neurona* in Marine Mammals and Opossums

Animal type	Animal ID^a^	Genetic marker analyzed									
		Microsatellite			snSAG3						
		sn7	sn3	sn9	239	506	507	508	509	735	1057
		(CA)n	(AT)n	(GT)n							
Marine Mammal	SL1	22	*	14	C	T	DEL	DEL	T	T	T
	SL1F^b^	22	11	14	*						
	SO1	*	12	*	*						
	HS1	*	*	*	*						
	HS2^c^	22	10	14	G	DEL	DEL	DEL	DEL	C	T
	HS3	*	*	*	*						
	HP1	23	11	14	C	T	A	T	A	T	C
	**HP2**	**22**	**11**	**14**	**C**	**T**	**A**	**T**	**A**	**T**	**C**
	HP3	25	-	-	G	T	A	T	A	T	C
	HP4	23	11	14	C	T	A	T	A	T	C
	PWSD1	22	10	14	G	DEL	DEL	DEL	DEL	C	C
	LBCD1	*	*	*	*						
Opossum	**OP1**	**22**	**11**	**14**	**C**	**T**	**A**	**T**	**A**	**T**	**C**
	OP2	21	11	14	C	T	A	T	A	T	C
	**OP3**	**22**	**11**	**14**	**C**	**T**	**A**	**T**	**A**	**T**	**C**

The nucleotide shown beneath the snSAG3 genetic markers indicates polymorphic sites among the *S. neurona* strains. DEL indicates deletions; asterisk (*) indicates no amplification. Bolded text denotes animals sharing genetic identity among examined loci for *S. neurona*

^a^SL = Sea Lion, SO = Sea Otter, PWSD = Pacific White-Sided Dolphin, HS = Harbor Seal, HP = Harbor Porpoise, LBCD = Long-Beaked Common Dolphin, OP = Opossum

^b^F detonates the neonatal fetus of the corresponding mammal

^c^HS2 matches genotype XIII described in Barbosa et al. (2015)

### Watershed and Groundwater Pathway Mapping

**Figure 2:**
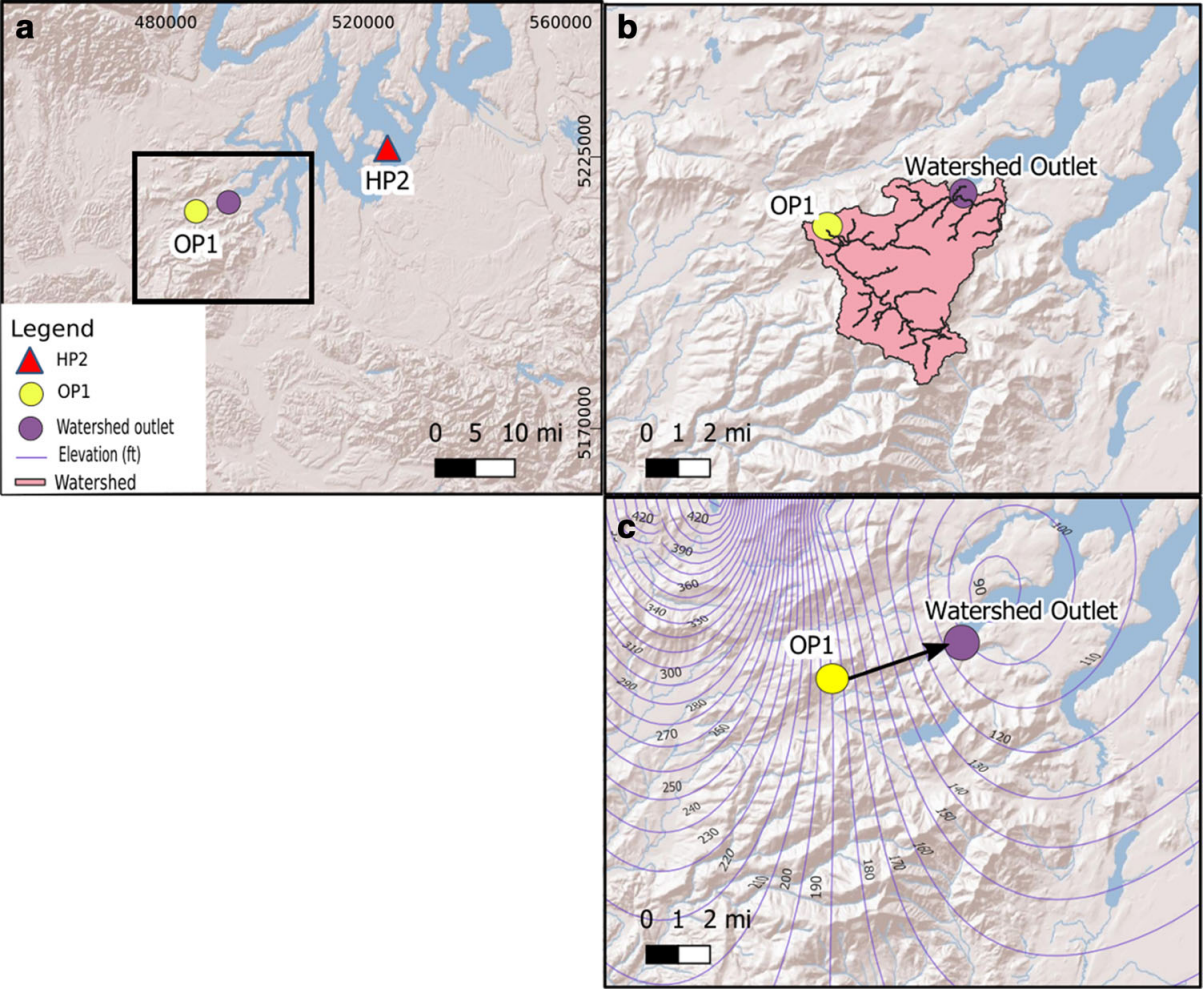
**a** Map depicting study region (inset) in Washington State, USA. **b** Watershed map with pink indicating the water flow from the site where an *S. neurona*-positive opossum (OP1) was collected to the Totten inlet (purple circle). This body of water is directly linked with the region where a harbor porpoise (HP2) carcass was shown to be infected with *S. neurona* that shared identical sequences among the four targeted loci. **c** Groundwater map depicting flow of groundwater (black arrow) from the sampling location of OP1 into coastal waters where HP2 had stranded.

Hydrological mapping was conducted for the two opossums (OP1 and OP2) that were shedding sporocysts bearing the same *S. neurona* genotype as HP2; no hydrological connection was observed between OP2 and HP2 (data not shown). For OP1, *S. neurona* sporocysts were estimated to travel toward the east in two pathways, via groundwater and surface water. Sporocysts entrained in surface water following rainfall events were likely to travel in overland runoff southward to a topographic low, eventually moving due east toward the Totten Inlet (Fig. 2), which is hydrologically connected to the water body in which HP2 stranded. The groundwater contour map showed a similar direction of water flow (Fig. 2). Sporocysts originating from OP1 feces that were not entrained in overland flow could infiltrate soil vertically to the groundwater table. Once sporocysts reached the water table, they would flow within the underground water gradient toward the east, and ultimately exit into the Totten Inlet.

## Discussion

This study demonstrates for the first time the presence of *S. neurona* in opossums in western Washington, with a prevalence of 9.4% actively shedding sporocysts. We have identified molecular linkages between *S. neurona* shed by terrestrial opossums and parasite genotypes in infected marine mammals in the Pacific Northwest in two ways. First, genetic identity was found between two opossums (OP1 and OP2) and a harbor porpoise (HP2) across four genetic markers (snSAG3, sn3, sn7, sn9). Second, in the first hydrological source-to-sea connection study of *S. neurona* in the Pacific Northwest, watershed and groundwater mapping demonstrated hydrological pathways connecting sporocysts shed on land with coastal waters where infected marine mammals have stranded. Thus, we provide molecular and hydrological support for the land-to-sea flow of *S. neurona* in the Pacific Northwest. Of note, the opossums included in this study were obtained opportunistically as road-killed animals, and thus the *S. neurona* prevalence presented here may not reflect a true population prevalence.

Rejmanek et al. (2009) have previously reported the prevalence of *S. neurona* in opossums from central California to be less than 6% based on molecular detection of sporocysts in GI scrapings or feces. In their study, multiple risk factors for infection were identified, including adult age, a non-coastal location and collection of samples between March and July (Rejmanek et al. 2009). As our current study included a more limited sample size of predominantly adult opossums and was performed on animals collected only between January and June, our ability to compare risk factors for seasonal and demographic variables was limited. Prevalence of *S. neurona* shedding in the eastern coast of the USA is reported to be higher at 14.9%–31.8% (Elsheikha et al. 2004; Dubey 2000). The lower prevalence on the west coast of the USA has been attributed to the more recent introduction of opossums to the region and therefore the parasite may not be as prevalent in this population (Rejmanek et al. 2009).

Through molecular characterization of *S. neurona* in central California, Rejmanek et al. (2010) demonstrated that opossums shed sporocysts that are molecularly identical to strains obtained from marine mammal tissues. Here, we report a similar potential molecular link as we identified two opossums (OP1 and OP2) and one harbor porpoise (HP2) that shared genetic identity across four genetic markers (snSAG3, sn3, sn7, sn9). We further investigated hydrological connections in the study area to evaluate if the physical flow of runoff from the terrestrial definitive hosts on land could be traced to the stranding locations of infected marine mammals. Watershed modeling has been used help predict movement of land-based pathogens from source to sea (VanWormer et al. 2016). The hydrological maps that we produced in this study suggest that *S. neurona* sporocysts shed in the feces of OP1 may travel via two pathways, in groundwater or surface water, both in an easterly direction toward the Totten Inlet (Fig. 2B). Transport of sporocysts through groundwater depends on a multitude of factors, including sporocyst charge and soil type (Dumètre et al. 2011). Closely related protozoal parasites such as *T. gondii* have been found in ground and surface water, thus demonstrating that fecally shed parasites similar in size and shape to *S. neurona* can infiltrate groundwater (Bahia-Oliveira et al. 2017). Both pathways could result in contaminated waters flowing into the Totten Inlet that hydrologically connects with the Carr Inlet, where HP2 had stranded.

While the projected hydrological transport pathways of sporocysts from the opossum to a stranded, infected marine mammal supports our molecular findings, there are factors that should be considered when investigating land-to-sea pathogen transmission. First, the stranding location of HP2 may differ from location of exposure; it is thus possible that infection has occurred several weeks earlier in a different coastal site, or even months to years earlier if the infection was chronic in nature. Further pathological examination to establish chronicity of protozoal infection in stranded marine mammals was beyond the scope of this study but should be investigated in the future. There is also the possibility that carcasses can drift post-mortem, and thus locations where their bodies were recovered may not represent location of death or exposure. Movement of sporocysts may also occur through paratenic hosts if those are mobile (e.g., fish). To date, the only lower tropic animals in which *S. neurona* has been detected are mussels (Michaels et al. 2016); however, studies investigating *S. neurona* in marine food webs are scarce.

Genetic characterization *S. neurona* based on the snSAG3 gene and microsatellite (MS) markers (sn3, sn7 and sn9) was chosen as they were previously reported to be more polymorphic among the 13 genetic markers analyzed by Rejmanek et al. (2010). Three of the markers (snSAG, sn3 and sn9) analyzed in this study were also included in a study by Barbosa et al. (2015), and the genetic profile at these markers in one harbor seal (HS2) was consistent with the novel *S. neurona* genotype XIII (Barbosa et al. 2015). In their study, Barbosa et al. (2015) identified the presence of genotype XIII in 12 marine mammals from the outer Washington coast: one harbor porpoise, one Steller sea lion and 10 Pacific harbor seals. Each of these 12 cases had marked to severe protozoal encephalitis, leading the authors to conclude that this genotype is likely to be a more virulent parasite strain (Barbosa et al. 2015). Our current finding of genotype XIII in a harbor seal (HS2) collected in 2017 indicates the persistence of this genotype within the sampled marine mammal population.

Molecular sequencing at the ITS1 gene showed that one harbor seal (HS7) was infected with *S. pinnipedi,* a novel *Sarcocystis* species that was implicated as a cause of necrotizing hepatitis and associated mortality in ringed seals, Pacific walrus (*Odobenus rosmarus divergens*), bearded seal (*Erignathus barbatus*), spotted seal (*Phoca largha*) and gray seals (*Halichoerus grypus)* (Haman et al. 2015). Previous work has suggested a close evolutionary relationship between *S. pinnipedi* and *S. canis*, which has been described in dogs and bears (Dubey et al. 2006a, b; Britton et al. 2019; Davies et al. 2011). *Sarcocystis pinnipedi* was initially isolated in gray seals after a mass mortality event in Nova Scotia, Canada in March 2012. This study is the first report of *S. pinnipedi* infection in a harbor seal and the identification of this pathogen in the northwest Pacific Ocean indicates that the range of *S. pinnipedi* is not restricted to the Atlantic or Arctic Oceans where it was first identified.

While the apparent prevalence of *S. neurona* in the tested marine mammals was 40% (*n* = 30), the population of animals included in this study was considered non-random based on the inclusion criteria previously defined in the methods section. Therefore, the overall prevalence of *S. neurona* in the general marine mammal population in this region cannot be estimated from this work. A similar report that tested for *S. neurona* infection in a non-random marine mammal population in the Pacific Northwest reported a higher prevalence of 61.5% (Gibson et al. 2011). Specifically, for sea otters in the Washington Olympic peninsula, *S. neurona* prevalence has been shown to be 67% using indirect fluorescent antibody testing (Burgess et al. 2020).

In the subset of marine mammal samples that was tested for the presence of both *S. neurona* and *T. gondii* DNA (*n* = 20) using the pan apicomplexan ITS1 primer set, *T. gondii* was present in 20% and *S. neurona* in 35% of animals. This finding supports other studies that noted a relative higher prevalence of *S. neurona* infections compared with *T. gondii* in marine mammals from the Pacific Northwest (Gibson et al. 2011; Thomas et al. 2007). Only one harbor porpoise (HP4) had a co-infection with both *S. neurona* and *T. gondii* (5% prevalence). Other studies have reported higher prevalence of co-infection, at 42% (Gibson et al. 2011) and 30.8% in sea otters (Thomas et al. 2007; White et al. 2018).

Of the four pregnant females with unborn fetuses included in this study, three tested positive for *S. neurona*, one of which was the aforementioned HP4 animal co-infected with *T. gondii*. Horizontal transmission is considered to be the primary route of *T. gondii* transmission*;* however, vertical transmission has also been documented and has been hypothesized to play an important role in the epidemiology of the parasite (Shapiro et al. 2016; Miller et al. 2008; Barbosa et al. 2015). Data from this study further support the occurrence of *T. gondii* vertical transmission in marine mammals, with the presence of one congenitally infected fetus. In contrast with *T. gondii,* there are more limited reports on *S. neurona* vertical transmission. These include congenital infections in a sea otter (Shapiro et al. 2016), harbor porpoise, Steller sea lion, pygmy sperm whale and five harbor seals (Barbosa et al. 2015). Of the three *S. neurona*-positive dams in the current study, vertical transmission was documented in one sea lion, SL1 and her in-utero fetus SL1F (prevalence of 33.3%, *n* = 3). Further investigations are needed to evaluate the importance of vertical transmission in the epidemiology of *S. neurona* in marine mammals.

An unexpected finding from sequence analysis at the ITS1-500 locus was identification of one opossum to be infected with *Sarcocystis* sp*. AGP-1.* The *AGP-1* species was previously identified in a client-owned 10-year-old female African gray parrot which was attacked by an opossum (*Delphis spp.*) in Costa Rica and died one year later (Dubey et al. 2006a, b). This strain was found to be genetically different from *S. neurona*; however, molecular and phylogenetic analyses by Dubey et al. (2016) have suggested that the opossum may serve as a definitive host for this species. To our knowledge, there have been no previous reports of *Sarcocystis* sp*. AGP-1* detection in GI scrapings from an opossum, nor have there been any previous reports of this species outside of Costa Rica. Thus, the results presented here provide the first report of a sequence consistent with *Sarcocystis sp. AGP-1 at the ITS1-500 locus* collected from an opossum. Further molecular characterization would be required to further investigate genetic similarity.

As the opossum is a non-native species to Washington, the introduction of this species is likely associated with the emergence of *S. neurona* in endemic wildlife, both terrestrial and marine (Barbosa et al. 2015). A similar link has been shown with the introduction of domesticated cats into Hawaii (USA), which likely resulted in the introduction of *T. gondii*. Subsequently, *T. gondii* associated mortality has been identified in a number of endangered species, both terrestrial and marine, including the critically endangered Hawaiian monk seal (*Neoonachus schauinislandi)* (Barbieri et al. 2016; Honnold et al. 2005). Similarly, the northern expanding range of opossums and associated introduction of *S. neurona* to the Pacific Northwest may have resulted in increased detrimental outcome to naïve marine wildlife.

## Conclusion

This study provides the first report of *S. neurona* in opossums from western Washington State, USA. Our results demonstrate molecular and hydrological evidence of transmission pathways of *S. neurona* from opossums to marine mammals in the Pacific Northwest, further supporting land-sea transmission of a protozoan parasite that can have detrimental impacts on marine wildlife. Investigations that identify specific temporal and spatial parameters associated with *S. neurona* infections in opossums may further assist targeted mitigation strategies for reducing the burden of *S. neurona* illness in susceptible hosts, including marine mammals.

## Supplementary Information

Below is the link to the electronic supplementary material.

Supplementary file1 (XLSX 61 KB)

Supplementary file2 (DOCX 20 KB)
