# Supplementary material for: Sarcocystis neurona Transmission from Opossums to Marine Mammals in the Pacific Northwest
Source: Ecohealth. 2021 Jul 2;18(1):84–94. doi: 10.1007/s10393-021-01536-w (PMC8367900; doi:10.1007/s10393-021-01536-w)
Supplement: Supplementary file 2 — Supplementary file2 (DOCX 20 KB) [file 10393_2021_1536_MOESM2_ESM.docx]

**Table S2**. Oligonucleotide primers used for molecular detection and characterization of *Sarcocystis* spp. in marine mammals and opossum. The ITS1 primer set coamplifies closely related apicomplexan parasites including *Toxoplasma gondii*.

| **Marker** | **Primer name** | **Oligonucleotide Sequence (5’ to 3’)** | **Reference** |
| --- | --- | --- | --- |
| ITS1 | ITS1Ext-F | TTCTCTTGTGTGTGCCCCTAC | Miller et al., 2009 |
|  | ITS1Ext-R | TGCGTCCTTCATCGTTGCGC |  |
|  | ITS1int-F | CAAAATGAACGTGTCTATGTGTGA |  |
|  | IST1int-R | GAGCCAAGACATCCATTGCT |  |
| ITS1-500 | ITS1-500ext-F | TTCTCTTGTGTGTGCCCCTAC | Miller et al., 2009 |
|  | IST1-500ext-R | TGCGTCCTTCATCGTTGCGC |  |
|  | IST1-500int-F | CAAAATGAACGTGTCTATGTGTGA |  |
|  | ISTI-500int-R | GAGCCAAGACATCCATTGCT |  |
| Sn7 | Sn7Ext-F | CGACAGTTCTCCCTGCTCTT | Rejmanek et al., 2010 |
|  | Sn7Ext-R | CATGCATCGATTTCTGATCG | Rejmanek et al., 2010 |
|  | Sn7Int-F | CCTGGTGATGAGTGAACTGC | Asmundsson and Rosenthal, 2006 |
|  | Sn7Int-R | TGGCCACCAGAGTCTTCTTT | Asmundsson and Rosenthal, 2006 |
| Sn3 | Sn3Ext-F | CAGGTCGTCCATTTTGGTCT | Asmundsson and Rosenthal, 2006 |
|  | Sn3Ext-R | GGATAAGGCACGCTTACAGG | Asmundsson and Rosenthal, 2006 |
|  | Sn3Int-F | CAGCAGGTCGTCCATTTTGG | Rejmanek et al., 2010 |
|  | Sn3Int-R | ACGTGCACGTGCATTGACAC | Rejmanek et al., 2010 |
| Sn9 | Sn9Ext-F | CTGCTGCTAGCGGACTCTCT | Wendte et al., 2010a |
|  | Sn9Ext-R | ACGCGCCTAAACGTGAATAG | Asmundsson et al. 2006 |
|  | Sn9Int-F | CGCCAAAAGACTCACAAACA | Asmundsson et al. 2006 |
|  | Sn9Int-R | Same as Sn9Ext-R | Asmundsson et al. 2006 |
| snSAG3 | snSAG3Ext-F | TCAAGGACGTTTTTCCCTGT | Wendte et al., 2010a |
|  | snSAG3Ext-R | CTCTGCATGCTGCAATGAAT |  |
|  | snSAGInt-F | CCCTGCCTTTCTGGTCTCTT |  |
|  | snSAGInt-R | TTCTCCCCAAAGACCATCTG |  |
